# Alteration of Mitochondrial DNA Copy Number and Increased Expression Levels of Mitochondrial Dynamics-Related Genes in Sjögren’s Syndrome

**DOI:** 10.3390/biomedicines10112699

**Published:** 2022-10-25

**Authors:** Giada De Benedittis, Andrea Latini, Serena Colafrancesco, Roberta Priori, Carlo Perricone, Lucia Novelli, Paola Borgiani, Cinzia Ciccacci

**Affiliations:** 1 Genetics Section, Department of Biomedicine and Prevention, University of Rome Tor Vergata, 00133 Rome, Italy; 2 Division of Rheumatology, Department of Clinical Internal, Anaesthesiologic and Cardiovascular Sciences, Sapienza University, 00133 Rome, Italy; 3 UniCamillus—Saint Camillus International University of Health Sciences, 00131 Rome, Italy; 4 Rheumatology Department of Medicine, University of Perugia, Piazzale Giorgio Menghini 1, 06129 Perugia, Italy

**Keywords:** Sjögren’s syndrome, mtDNA, mitochondrial dynamics, oxidative stress

## Abstract

Sjögren’s syndrome (SS) is a chronic autoimmune multifactorial disease characterized by inflammation and lymphocytic infiltration of the exocrine glands. Several studies have highlighted the involvement of oxidative stress in this pathology, suggesting that it could induce mitochondrial dysfunctions. Mitochondria could have a role in inflammatory and immune processes. Since the mitochondrial DNA (mtDNA) copy number could change in response to physiological or environmental stimuli, this study aimed to evaluate possible alterations in the mtDNA copy number in SS. We have analyzed the amount of mtDNA in the peripheral blood of 74 SS patients and 61 healthy controls by qPCR. Then, since mitochondrial fusion and fission play a crucial role in maintaining the number of mitochondria, we investigated the expression variability of the genes most commonly involved in mitochondrial dynamics in a subgroup of SS patients and healthy controls. Interestingly, we observed a highly significant decrease in mtDNA copies in the SS patients compared to healthy controls (*p* = 1.44 × 10^−12^). Expression levels of mitochondrial fission factor (*MFF*), mitofusin-1 (*MFN1*), and mitochondrial transcription factor A (*TFAM*) genes were analyzed, showing a statistically significant increase in the expression of *MFF* (*p* = 0.003) and *TFAM* (*p* = 0.022) in the SS patients compared to healthy controls. These results give further insight into the possible involvement of mitochondrial dysfunctions in SS disease.

## 1. Introduction

Sjögren’s syndrome (SS) is a chronic autoimmune disease characterized by inflammation and lymphocytic infiltration of the exocrine glands, particularly the lacrimal and salivary glands [1]. It is considered a multifactorial pathology with different factors contributing to its development: underlying genetic predisposition, epigenetic mechanisms, and environmental factors [2]. 

Over the last years, many different research groups have demonstrated that inflammation is often associated with impaired mitochondrial function [3,4,5] in different diseases, suggesting that mitochondria could play a role in inflammatory processes. Among autoimmune diseases, an association with mitochondrial dysfunctions has been described in systemic lupus erythematosus [6], multiple sclerosis [7], rheumatoid arthritis [8] and type 1 diabetes [9].

Mitochondria are the principal source of energy that cells need for normal function. Energy, in the form of ATP (adenosine triphosphate), is generated by the passage of electrons from donors at a lower redox potential to acceptors at a higher one. During this process, 2% of molecular oxygen is not completely reduced to water, and, therefore, reactive oxygen species (ROS) can be produced [10]. When mitochondrial function is impaired, an increase in the production of ROS is observed, and, consequently, a condition of strong oxidative stress (OS) is induced. Several autoimmune diseases are characterized by an increased expression of OS markers and antimitochondrial antibodies [11,12]. The involvement of oxidative stress is known in patients with SS, in which we can observe increased levels of OS markers in both plasma and saliva [13,14]. Furthermore, 3–27% of SS patients produce antimitochondrial antibodies, which may predict the development of primary biliary cholangitis [15,16]. 

Mitochondria are highly dynamic organelles, and the preservation of their number and morphology is regulated by a balance between fission and fusion cycles. Both mitochondrial fusion and fission are essential in mammals, and these processes are called “mitochondrial dynamics”. This equilibrium can be shifted to one or the other process in response to several factors, including the cell’s metabolic state or OS. The perturbation of mitochondrial dynamics could alter the physiological mitochondrial function. While the reduction in mitochondrial fusion induces mitochondrion fragmentation, the reduction in mitochondrial fission leads to elongated and excessively interconnected mitochondrion [17]. 

Mitochondrial fusion is the mechanism that allows two adjacent mitochondria to merge and form a single larger one, and it is typically implemented when the cell needs fewer mitochondria. This process is directed by proteins that bind and promote membrane fusion, including mitofusin-1 (MFN1), a transmembrane GTPase responsible for the fusion of the outer membrane. Mitochondrial fusion plays an important role in mitochondrial quality control because it alleviates OS by combining the content of partially damaged mitochondria [18].

On the contrary, mitochondrial fission is the mechanism that generates two mitochondria starting from one; it is driven by proteins that first direct the constriction of membranes and then mitochondrial splitting. Among these proteins, mitochondrial fission factor (MFF) is a tail-anchored protein of the mitochondrial outer membrane that acts as the receptor for Drp1 (dynamin-related protein-1), a cytosolic GTPase that tends to oligomerize. This process is not only essential for mitochondrial duplication and biogenesis but also for the clearance of damaged mitochondria during high levels of cellular stress [18]. 

Moreover, each mitochondrion has multiple copies of mitochondrial DNA (mtDNA). It is highly vulnerable to OS, which can exert both qualitative and quantitative changes. Mitochondrial transcription factor A (TFAM) is essential for the preservation and the transcription of mtDNA during mitochondrial biogenesis; it is a high mobility group domain protein that is localized in mitochondria but encoded by nuclear genes [19]. It has been reported that *TFAM* overexpression accelerates the recovery of mtDNA levels after oxidative stress damage in rats [20]. Accordingly, this factor seems to protect and prevent damage from ROS, as well as regulate the mtDNA copy number [21,22]. 

Recently, a transmission electron microscopy study has revealed alterations in the mitochondria of salivary gland epithelial cells in SS patients [23]. However, there are few studies regarding the dysregulation of mitochondrial dynamics in SS patients, and none has reported data related to the mtDNA copy number. 

In light of these considerations, we aimed to evaluate the amount of mtDNA in peripheral blood mononuclear cells (PBMCs) of patients with SS and healthy subjects to investigate possible alterations. Afterward, we analyzed, in two subgroups of patients and control subjects, the expression variability of the most common genes involved in mitochondrial dynamics: *MNF1*, *MFF*, and *TFAM*.

## 2. Materials and Methods

### 2.1. Patients Recruitment

Seventy-four patients with SS, diagnosed according to the 2016 ACR-EULAR Classification Criteria [24], were consecutively enrolled from the Sjögren’s Clinic of Sapienza University of Rome. The study protocol included a complete physical examination and blood draw. The clinical and laboratory data were collected in a standardized filled form, including demographics, past medical history, date of diagnosis, comorbidities, and previous/concomitant treatments. Sixty-one age-, sex-, and ethnicity-matched healthy subjects were enrolled, as controls, at the University of Rome “Tor Vergata”. Written informed consent was obtained from each participant, and the ethical committee of Sapienza University of Rome approved the study design (approval number 4688/2018). Peripheral blood samples from all patients and controls have been collected and stored at −20 °C until usage.

### 2.2. Mitochondrial DNA Copy Number Evaluation

Nuclear and mitochondrial DNA was extracted from PBMCs using a Qiagen blood DNA mini kit. An mtDNA copy number analysis was performed as described by Rooney [25]. Primers amplifying a nuclear DNA region (hemoglobin subunit beta [*HGB*]) and an mtDNA region (NADH dehydrogenase subunit 1, [*ND1*]) were available in the literature [26]. Five ng of total cellular DNA was used as input for quantitative PCR (qPCR), and the reactions were performed with a 7500 Real-Time PCR System (Applied Biosystems, Foster City, CA, USA). Each reaction was performed in triplicate. The Ct values for the nuclear HGB gene and mitochondrial ND1 gene were concurrently determined in each sample during the same qPCR run. The mitochondrial copy number in the leukocytes of each subject was calculated by the equation (2 × 2^(Ct.(HGB)−Ct.(ND1))^) [26].

### 2.3. mRNA Isolation and Expression Analysis

The expression levels of *MNF1*, *MFF*, and *TFAM* were analyzed in a subgroup of 27 SS patients and 15 healthy controls (CTRLs), randomly selected from the first cohort for the expression study. Total RNA was isolated from PBMCs using the TRIzol reagent (Ambion, CA, USA) protocol, followed by reverse transcription using the High Capacity cDNA Reverse Transcription Kit (Applied Biosystems, Waltham, MA, USA). Expression analysis was performed by quantitative RT-polymerase chain reaction (SYBR Green Assay, Applied Biosystems) using the 7500 Real-Time PCR System (Applied Biosystems, Foster City, CA, USA). The primers used to detect gene expression are listed in Supplementary Table S1. Each sample was analyzed in triplicate, and each assay was run with an endogenous control (β-Actin) to standardize the results. Relative expression levels were calculated using the 2^−ΔΔCt.^ method, and data were reported as mean values ± standard deviation.

### 2.4. Statistical Analysis

The analysis of variance (ANOVA) test was used to compare mean mtDNA copy number levels and gene expression data among the different phenotypic groups. A *p*-value ≤ 0.05 was considered significant in all the completed tests. The receiver operating characteristic (ROC) curve analysis was performed to evaluate the differentially expressed genes’ ability to discriminate the two groups, patients and controls. All statistical analyses were performed by the SPSS program, version 19 (IBM Corp, Armonk, NY, USA).

## 3. Results

Clinical and laboratory data of the 74 enrolled patients are presented in Table 1. 

In line with the epidemiologic data [27], 92.2% of patients were females, with a mean age of 59.5 years and a mean age at diagnosis of 51.1 years. Lymphoproliferative complications were reported in 10.8% of cases (non-Hodgkin lymphoma). Additionally, 21.6% of patients presented salivary gland swelling. For the control group, 80.0% of the subjects were females, with a mean age of 59.3 years.

**Figure 1:**
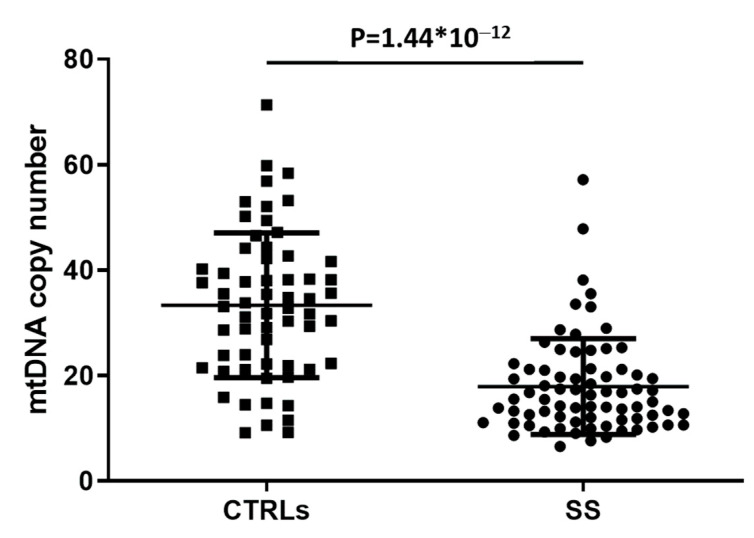
Comparison of mtDNA copy number between healthy controls (CTRLs) and patients with Sjögren syndrome (SS). The mtDNA copy number in leukocytes of each subject was calculated by the equation (2 × 2^(Ct.(HGB)−Ct.(ND1))^).

The amount of mtDNA was analyzed in the peripheral blood of 74 SS patients and 61 healthy CTRLs. As shown in Figure 1, the mean count of mtDNA copy number was significantly lower in patients with SS compared to the healthy subjects (*p* = 1.44 × 10^−12^). 

We verified whether the mtDNA copy number was associated with patients’ clinical characteristics, such as salivary gland swelling, arthritis, lymphoma, and autoantibodies seropositivity. However, no significant association emerged. Furthermore, the mtDNA content was not correlated with age and sex in SS patients.

**Figure 2:**
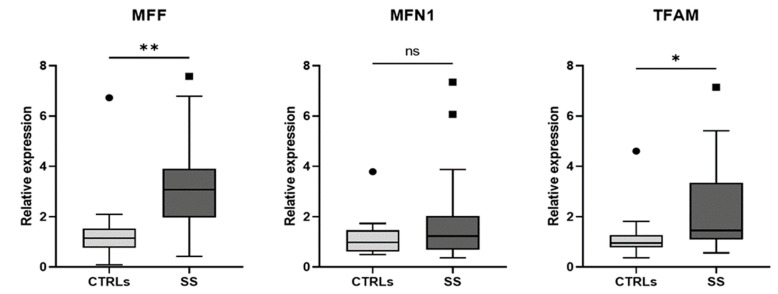
Comparisons of gene expression levels between healthy controls (CTRLs) and patients with Sjögren syndrome (SS). ** *p* = 0.003; * *p* = 0.022; ns = nonsignificant. Mitochondrial fission factor (*MFF*), mitofusin-1 (*MFN1*), and mitochondrial transcription factor A (*TFAM*). The endogenous control used is β-Actin.

In light of these data, we subsequently analyzed the expression levels of genes involved in mitochondrial dynamics in two subgroups, randomly selected, of 27 SS patients and 15 healthy CTRLs. Clinical and laboratory data of these 27 SS patients are presented in Table 1. Expression levels of *MFF*, *MFN1* and *TFAM* genes in SS patients and healthy CTRLs are shown in Figure 2. 

As reported, a significant increase in the expression of *MFF* was observed in SS patients compared to healthy CTRLs (*p* = 0.003); on the contrary, no significant difference in the expression of *MFN1* was observed. Moreover, a significant increase in the expression of *TFAM* was observed in SS patients compared to healthy CTRLs (*p* = 0.022). No significant correlation emerged between the expression levels of these genes and specific clinical characteristics. 

**Figure 3:**
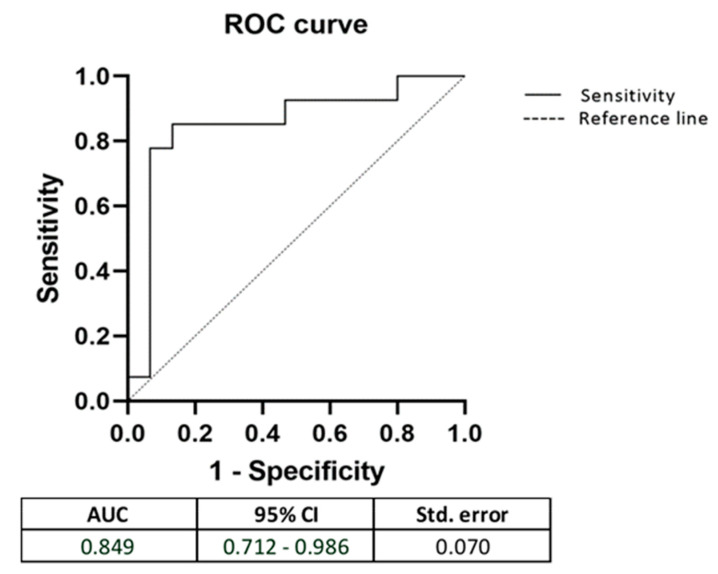
Receiver operator characteristic curves, including MFF and TFAM. AUC: Area under the curve; CI: Confidential interval; Std: Standard deviation.

Since *MFF* and *TFAM* showed a significant increase in expression levels in SS patients, we performed the ROC curve analysis to evaluate the capacity of these genes to predict the subjects at higher risks of developing SS (Figure 3). 

The results demonstrated that the area under the ROC curve (AUC) for the model, including both genes, was 0.849 with 85% sensitivity and 87% specificity.

## 4. Discussion

Oxidative stress induces mitochondrial dysfunctions accompanied by deregulation between mitochondrial fission and fusion. These alterations are typical in the pathogenesis of several diseases, including inflammatory autoimmune diseases [5]. Recent studies have indicated the significance of OS influence in the disease process of SS. Data in the literature have described increased OS in SS patients [13,14]. Additionally, a recent transmission electron microscopy study has reported alterations in the mitochondria of salivary gland epithelial cells [23].

Since mtDNA copy numbers could change in response to physiological or environmental stimuli, as the increased OS, we have decided to evaluate the mtDNA copies in a cohort of SS patients.

Our study observed that the mean count of mtDNA copy number was significantly decreased in PBMCs of SS patients compared to healthy CTRLs. This data is in accordance with the hypothesis that increased OS may contribute to alterations in the number of mitochondria, as well as their mtDNA copy number [28]. 

Since mitochondrial fusion and fission play a crucial role in maintaining the number of mitochondria that could be altered when cells experience a condition of severe OS, we have investigated the expression variability of the genes most commonly involved in mitochondrial dynamics. We conducted our analysis in a randomly selected subgroup of 27 SS patients and 15 healthy CTRLs. We found that SS patients have higher levels of mRNA expression for the three mitochondrial-related genes, compared with the controls, reaching a statistical significance for *MFF* and *TFAM* genes.

The mitochondrial fission receptor MFF is a crucial factor for mitochondrial recruitment of Drp1 [29], and it has been reported that its overexpression leads to mitochondrial fragmentation, whereas its downregulation induces mitochondria elongation [30]. Furthermore, mitochondrial fission is essential during conditions of high cellular stress because it is responsible for the clearance of damaged mitochondria [18]. Indeed, functional studies have demonstrated that oxidized glutathione (GSSG), an important cellular stress indicator, strongly induces mitochondrial hyper-fusion in an Mfn-dependent manner [31]. We could hypothesize that its expression increases in these patients in response to the high levels of OS described in SS patients. 

*MFN1* encodes for a transmembrane GTPase responsible for the outer mitochondrial membrane [32], but it is also involved in controlling the number of healthy mitochondria and decreasing the ROS [33]. We found that its expression increased in the SS patients analyzed compared to the control subjects, although this difference does not reach statistical significance. We could suppose that the increase in MFN1 is due to its ability to decrease the amount of ROS. 

In light of these data, we could hypothesize that the OS in SS patients causes a perturbation both in mitochondrial fission and fusion through increased expression of *MFF* and *MFN1*, resulting in mitochondrial fragmentation, as recently confirmed in salivary gland epithelial cells by Barrera et al. [23]. This fragmentation could contribute to altering the physiological mitochondrial function. 

However, in contrast with our results, a recent study reported that *MFF* and *MFN1* are downregulated in labial salivary gland samples of Chinese SS patients stratified in different stage groups [34]. These conflicting results could be due to the different tissue investigated in the Chinese study and the particular severe phenotype analyzed.

Finally, we found that the expression level of the *TFAM* gene is higher in SS patients compared to healthy CTRLs. TFAM is a crucial mitochondrial transcription factor, and its main role is to regulate the mtDNA copy number [21]. TFAM has another role: it may protect and prevent mtDNA damage from ROS [22]. In fact, TFAM plays a protective role against β-amyloid induced oxidative damage in human neuroblast cells and astrocytes [35,36]. Furthermore, it has been described that an intense overexpression of *TFAM* results in the suppression of mitochondrial gene expression and ultrastructural changes of mitochondria in different tissues [37]. Thus, we could suppose that the increased TFAM levels in SS patients are due to a compensatory response carried out to restore mtDNA and, simultaneously, to protect it from oxidative damage.

Finally, considering the aberrant expression of *MFF* and *TFAM* in PBMCs of SS patients, we used a ROC curve to evaluate the ability of these genes to discriminate SS subjects from the control group. We found that the expression levels of *MFF* and *TFAM* show high sensitivity and specificity in distinguishing SS patients from healthy CTRLs. However, even if interesting, this result needs further confirmation in larger cohorts and further study to understand its functional role. 

To our knowledge, this study is one of the first to compare mtDNA copy numbers in the peripheral blood of patients with SS and healthy CTRLs. However, our results contrast with the ones reported in a previously published paper, which showed an increase in mtDNA copy number in a group of Chinese SS female patients compared with controls [38]. These conflicting results suggest the need to replicate this evaluation in other populations and in larger cohorts.

Our research has some limitations: he lack of data regarding the measurement of oxidative stress markers in our SS patients is related to insufficient available material; the impossibility of performing a correlation analysis between the mtDNA copies and the expression levels of *MNF1*, *MFF*, and *TFAM* genes, because we have collected a second round of blood draw in the subgroup of 27 SS patients at a later time, specifically used for the RNA experiments; hte absence of functional studies to understand the molecular mechanisms underlying our results. Moreover, additional data are necessary to clarify if the mitochondria involvement is an effect of Sjögren’s syndrome or a factor that contributes to the onset of the disease.

In conclusion, our data show a quantitative alteration of mtDNA in patients with SS. Moreover, we describe a significant increase in expression levels of genes involved in mitochondrial dynamics in SS patients, specifically in mitochondrial fission. These results suggest a possible involvement of both OS and mitochondrial dysfunctions in the pathogenesis of SS disease. If confirmed in other cohorts and eventually supported by functional studies, these results could provide additional support to the important role played by mitochondrial remodeling and OS in the development of SS.

## Figures and Tables

**Table 1:** Clinical and laboratory data of the SS patients.

	SS (*n* = 74)	SS Subgroup (*n* = 27)
Sex (% of females)	92.2	88.5
Age (mean ± SD)	59.5 ± 11.09	58.79 ± 10.33
Age at diagnosis (mean ± SD)	51.1 ± 11.65	54.14 ± 9.76
Xerophthalmia (%)	98.6	96.4
Xerostomia (%)	90.5	85.7
Salivary gland swelling (%)	21.6	3.6
Arthritis (%)	14.9	14.3
Lymphoma (%)	10.8	7.1
ANA (%)	91.9	82.1
Anti-SSA (%)	86.5	64.3
Anti-SSB (%)	60.8	53.6
Hypergammaglobulinemia (%)	41.9	39.3
Rheumatoid factor (%)	41.1	23.1
Leukopenia (%)	23.0	28.6
Hypocomplementemia (%)	5.5	14.3
Monoclonal component (%)	11.1	14.8
Cryoglobulins (%)	4.2	0

Quantitative data are expressed as mean and standard deviation (SD); dichotomous data are expressed as a percentage. ANA (antinuclear antibodies) were considered positive if titer ≥ 1160; anti-SSA (anti-SS-related antigen A autoantibodies), anti-SSB (anti-SS-related antigen B autoantibodies), and rheumatoid factor were considered positive according to the cutoff of the reference laboratory; hypergammaglobulinemia was diagnosed if total Ig ≥ 20% of total proteins; leukopenia was diagnosed if WBC < 4000/mm^3^; hypocomplementemia was diagnosed if C3 < 80 mg/dL and/or C4 < 15 mg/dL.

## Data Availability

Data are available on request.

## Supplementary Materials

The following supporting information can be downloaded at: https://www.mdpi.com/article/10.3390/biomedicines10112699/s1, Table S1: Primer sequences.
