# Supplementary material for: Alteration of Mitochondrial DNA Copy Number and Increased Expression Levels of Mitochondrial Dynamics-Related Genes in Sjögren’s Syndrome
Source: Biomedicines. 2022 Oct 25;10(11):2699. doi: 10.3390/biomedicines10112699 (PMC9687724; doi:10.3390/biomedicines10112699)
Supplement: Supplementary file 1 [file biomedicines-10-02699-s001.zip › biomedicines-1945463-supplementary.pdf]

Table S1. Primer sequences.

GENE	PRIMER SEQUENCE (5′ --> 3′)
Mitochondrial Fission Factor (MFF)	FW: GTGTGATAATGCAAGTTCCGG<br>RV: GTTTTCAGTGCCAGGGGT
Mitofusin-1 (MFN1)	FW: AGACAAGCTTTCCATCATTGGT<br>RV: TTGATAACAGAGCTCTTCCCAC
Mitochondrial Transcription Factor A (TFAM)	FW: TGTGTATTTACCGAGGTGGTTT<br>RV: AACGCTGGGCAATTCTTCTA
β-Actin	FW: AAGATGACCCAGATCATGTTTGAGACC<br>RV: ATCCTGCGTCTGGACCTGGCGT
